# Reduced Fitness Costs of *mcr-1.2* Compared to Mutated *pmrB* in Isogenic Colistin-Resistant KPC-3-Producing Klebsiella pneumoniae

**DOI:** 10.1128/mSphere.00551-19

**Published:** 2019-11-06

**Authors:** Cesira Giordano, Adrian Klak, Simona Barnini, Monika A. Chlebowicz, Mariacristina Menconi, John W. Rossen, Alexander W. Friedrich, Erik Bathoorn

**Affiliations:** a SD Ospedaliera di Microbiologia, Azienda Ospedaliero-Universitaria Pisana, Pisa, Italy; b University of Groningen, University Medical Center Groningen, Department of Medical Microbiology, Groningen, Netherlands; c UO Oncoematologia Pediatrica, Azienda Ospedaliero-Universitaria Pisana, Pisa, Italy; JMI Laboratories

**Keywords:** colistin, fitness cost, *mcr-1.2*, molecular epidemiology

## Abstract

In the present study, we provide the results of a detailed genomic analysis and the growth characteristics of a colistin-resistant KPC-3-producing Klebsiella pneumoniae sequence type 512 (ST512) isolate (the colR-KPC3-KP isolate) with a mutated *pmrB* and isogenic isolates of colR-KPC3-KP with *mcr-1.2* isolated from an immunocompromised patient. From 2014 to 2017, four colR-KPC3-KP isolates were detected in rectal swab samples collected from a pediatric hematology patient at the Azienda Ospedaliero-Universitaria Pisana in Pisa, Italy. Whole-genome sequencing was performed by MiSeq sequencing (Illumina). Growth experiments were performed using different concentrations of colistin. The growth lag phases both of an isolate harboring a deletion in *pmrB* and of clonal variants with *mcr-1.2* were assessed by the use of real-time light-scattering measurements. In the first isolate (isolate 1000-*pmrB*Δ, recovered in September 2014), a 17-nucleotide deletion in *pmrB* was detected. In subsequent isolates, the *mcr-1.2* gene associated with the plasmid pIncX4-AOUP was found, while *pmrB* was intact. Additionally, plasmid pIncQ-AOUP, harboring aminoglycoside resistance genes, was detected. The growth curves of the first three isolates were identical without colistin exposure; however, at higher concentrations of colistin, the growth curves of the isolate with a deletion in *pmrB* showed longer lag phases. We observed the replacement of mutated colR-KPC3-KP *pmrB* by isogenic isolates with multiple resistance plasmids, including *mcr-1.2-*carrying pIncX4, probably due to coselection under gentamicin treatment in a patient with prolonged colR-KPC3-KP carriage. The carriage of these isolates persisted in follow-up cultures. Coselection and the advantages in growth characteristics suggest that the plasmid-mediated resistance conferred by *mcr* has fewer fitness costs in colR-KPC3-KP than mutations in chromosomal *pmrB*, contributing to the success of this highly resistant hospital-adapted epidemiological lineage.

**IMPORTANCE** Our study shows a successful prolonged human colonization by a colistin-resistant Klebsiella pneumoniae isolate harboring *mcr-1.2*. An intense antibiotic therapy contributed to the maintenance of this microorganism through the acquisition of new resistance genes. The isolates carrying *mcr-1.2* showed fewer fitness costs than isogenic isolates with a *pmrB* mutation in the chromosome. Coselection and reduced fitness costs may explain the replacement of isolates with the *pmrB* mutation by other isolates and the ability of the microorganism to persist despite antibiotic treatment.

## Abstract (precis)

Our study shows a successful prolonged human colonization by a colistin-resistant Klebsiella pneumoniae isolate harboring *mcr-1.2*. An intense antibiotic therapy contributed to the maintenance of this microorganism through the acquisition of new resistance genes. The isolates carrying *mcr-1.2* showed fewer fitness costs than isogenic isolates with a *pmrB* mutation in the chromosome. Coselection and reduced fitness costs may explain the replacement of isolates with the *pmrB* mutation by other isolates and the ability of the microorganism to persist despite antibiotic treatment.

## INTRODUCTION

Colistin is a last-resort therapeutic option effective in the treatment of bacterial infections caused by multidrug-resistant (MDR) Gram-negative bacteria, particularly carbapenem-resistant Klebsiella pneumoniae, Acinetobacter baumannii, and Pseudomonas aeruginosa strains. These nosocomial pathogens, the commonest pathogens found in bacteremia and ventilator-associated pneumonia in critically ill patients, are evolving toward pan-resistance. The mortality rate, particularly among those with carbapenemase-producing K. pneumoniae (CP-KP) infections, may reach up to 75% and is attributed both to the virulence of the pathogen and to the lack of an effective antimicrobial therapy (1). Together with fosfomycin, tigecycline, and some recently introduced beta-lactam–beta-lactamase inhibitor combinations, e.g., ceftazidime-avibactam (2), colistin is considered one of the few available treatments against infections caused by MDR Gram-negative bacteria.

In the last few years, colistin resistance has emerged worldwide among patients previously treated with colistin. In addition, colistin resistance has also been described in humans without colistin therapy and in the absence of clonal transmission (3). Furthermore, not only does the clonal transmission of resistant strains with chromosomal mutations play an important role in the spread of colistin resistance among CP-KP bacteria, but also the horizontal dissemination of plasmids carrying insertion sequences associated with colistin resistance contributes to high resistance rates (4).

The most common molecular mechanism of colistin resistance is the modiﬁcation of the lipopolysaccharide (LPS), which is a mechanism analogous to that observed in bacteria with intrinsic resistance to polymyxins (5). Most often, resistance mechanisms are chromosomally encoded, affecting genes involved in the modiﬁcation of the LPS, e.g., the *pmrHFIJKLM* operon and the *pmrE* gene, the two-component systems PmrA/PmrB and PhoP/PhoQ, the *crrAB* operon, and the *mgrB* gene. The plasmid-associated colistin resistance gene *mcr-1* was first described in 2016 (6). Since its discovery, several variants of *mcr-1* have been described (7). The spread of plasmids harboring resistance genes into human-adapted pathogens through horizontal gene transfer generally represents a major public health risk. In addition, the detection of plasmids with *mcr-1* in successful nosocomial lineages is a cause of great concern, since this imposes a risk of patient-to-patient transmission, resulting in hospital outbreaks. For CP-KP bacteria, the early detection of carriage enables health care facilities to contain the spread through patient isolation and the implementation of measures of infection control (8). The colonization of patients with CP-KP bacteria may persist for months to years after detection of the first positive culture (9). To the best of our knowledge, there are no data regarding the duration of carriage of pathogens harboring *mcr* in humans.

The acquisition of resistance generally goes together with fitness costs, and this is also the case if the resistance is acquired by horizontal gene transfer. Resistance mediated by plasmids may carry a fitness cost reduced from that of resistance mediated by chromosomal mutations, since the costs can be reduced by compensatory mutations either in the plasmid or in the chromosomal DNA (10). In addition, plasmids may carry multiple resistance genes, which allows for the coselection of successful lineages of plasmids carrying genes for resistance to multiple antibiotics under the stress of treatment with various antibiotics. The analysis of fitness costs is often assessed by *in vitro* experiments using laboratory strains. Analysis of the fitness of isogenic isolates from the same patient that differ only by their resistance mechanisms is of importance to verify the robustness of study results with laboratory strains (11).

In the present study, we provide the results of a detailed genomic analysis of a colistin-resistant KPC-3-producing Klebsiella pneumoniae isolate (the colR-KPC3-KP isolate) with a mutated *pmrB* gene isolated from an immunocompromised pediatric patient. In the follow-up period of 3 years, isogenic isolates harboring different plasmids carrying various resistance genes, including *mcr-1.2*, as well as *strAB*, conferring colistin and aminoglycoside resistance, respectively, were detected. The duration of carriage, plasmid diversification, changes to the resistome, and growth characteristics of the colR-KPC3-KP isogenic isolates were assessed.

### Case description.

The patient was a child with acute lymphoblastic leukemia first admitted to hospital in August 2014. In September 2014, a rectal swab specimen was positive for colR-KPC3-KP (isolate 1000-*pmrB*Δ), and the patient underwent an oral gentamicin regimen (120 mg per day) for gut decontamination, combined with oral trimethoprim-sulfamethoxazole (800/160 mg twice daily 2 times per week) and fluconazole (100 mg per day) therapy. The patient was discharged after 10 days with scheduled clinical and hematological control visits. In October 2014, rectal swab specimens were still positive for colR-KPC3-KP. In November 2014, the patient was hospitalized for neutropenic fever caused by colR-KPC3-KP (isolate 1041-*mcr*) and treated with oral gentamicin (120 mg per day), trimethoprim-sulfamethoxazole (2 times per week), and nystatin (oral suspension 3 times per day). In December 2014, rectal swab specimens were still positive for colR-KPC3-KP (isolate 1074-*mcr*/pIncX3Δ). In January 2015, the patient continued the same antibiotic regimen described above until the end of February 2015. Then, the oral gentamicin regimen was administered for treating further CP-KP colonization. The child was never treated with colistin in 2014. In September 2015 (when isolate 1140-*mcr* was recovered), the patient was hospitalized for 10 days for neutropenic fever caused by colR-KPC3-KP and was treated with gentamicin intravenously (160 mg per day), meropenem intravenously (1,000 mg 3 times per day), colistin intravenously (3,000,000 U for the first day and then 1,700,000 U 2 times per day), nystatin (oral suspension 3 times per day), and fluconazole (oral, 100 mg per day). Cultures of rectal swab specimens were negative for colR-KPC3-KP beginning in October 2015. Overall, the patient was colonized with colR-KPC3-KP for 372 days and was treated with oral gentamicin for 382 days. In 2016, rectal swab specimens collected monthly were negative for colR-KPC3-KP; the last negative rectal swab specimen was collected in March 2017.

## RESULTS

### Characteristics of isolates.

**FIG 1:**
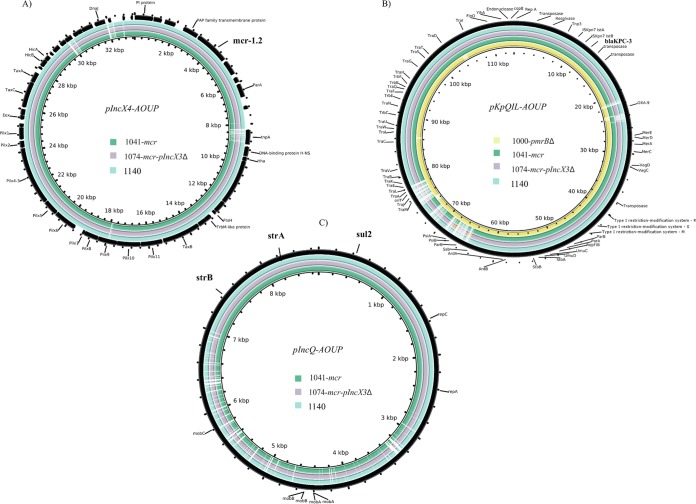
Comparison of the genomes of the pIncX4-AOUP plasmids (A), pKpQIL-AOUP plasmids (B), and pIncQ-AOUP plasmids (C) in colR-KPC3-KP isolates. Isolate 1000-*pmrB*Δ was collected in September 2014, isolate 1041-*mcr* was collected in November 2014, isolate 1074-*mcr*/pIncX3Δ was collected in December 2014, and isolate 1140-*mcr* was collected in September 2015. The black outer ring represents the reference plasmid.

An overview of the characteristics of the four isolates is presented in Table 1. In the first isolate (isolate 1000-*pmrB*Δ), a 17-nucleotide deletion in *pmrB* from nucleotide positions 386 to 402 was detected and associated with colistin resistance. In isolates 1041-*mcr*, 1074-*mcr*/pIncX3Δ, and 1140-*mcr* from subsequent cultures, this mutation was not present. In these isolates, *mcr1.2* associated with the pIncX4-AOUP plasmid (∼30 kbp) was detected (Fig. 1A). No mutations in the *mgrB*, *phoP*, *phoQ*, and *pmrA* genes with the predicted impact on biological functions were found in any isolate. All isolates carried the carbapenemase gene *bla*_KPC-3_, localized on the pKpQIL-AOUP plasmid (∼113 kbp) in a Tn*4401a* transposon (Fig. 1B). In addition, the *bla* genes encoding OXA-9 and SHV-11 were detected. A number of aminoglycoside resistance genes were found in all the isolates, namely, *aac(6′)-Ib*, *aadA2*, and *aph(3′)-I*. Also, the gene *aac(6′)Ib-cr*, responsible for low-level resistance both to fluoroquinolones and to aminoglycosides, was detected. In isolates 1041-*mcr*, 1074-*mcr*/pIncX3Δ, and 1140-*mcr*, other genes implicated in aminoglycoside resistance were found, in particular, the aminoglycoside acetyltransferase genes *aac(3)-IId* and *aadA5* and the aminoglycoside phosphotransferase genes *strA* and *strB*. The aminoglycoside resistance genes *strAB* and the gene *sul2*, involved in trimethoprim-sulfamethoxazole resistance, were localized on the newly acquired pIncQ-AOUP plasmid (∼9 kbp) (Fig. 1C). Both pIncQ-AOUP and pIncX4-AOUP were newly acquired in isolates 1041-*mcr*, 1074-*mcr*/pIncX3Δ, and 1140-*mcr*. Plasmid pIncX3-AOUP was found in isolates 1000-*pmrB*Δ and 1041-*mcr* and was subsequently lost. The patterns of susceptibility to antibiotics are presented in Table 2. All four isolates had MICs for colistin of >8 mg/liter and were resistant to meropenem (MIC = 32 mg/liter) and ertapenem (MIC > 1 mg/liter). The susceptibility to imipenem was intermediate (MIC = 8 mg/liter) for isolate 1000-*pmrB*Δ and resistant (MIC = 16 mg/liter) for the other three isolates. Isolate 1000-*pmrB*Δ was susceptible to fosfomycin, gentamicin, and tigecycline. Isolates 1041-*mcr*, 1074-*mcr*/pIncX3Δ, and 1140-*mcr* were also susceptible to fosfomycin and tigecycline but resistant to gentamicin, with MIC values being above 4 mg/liter.

**TABLE 1:** Characteristics of colR-KPC3-KP isolates and genes associated with antimicrobial resistance^*a*^

Isolate	Isolation date (day/mo/yr)	Day	MLST ST	Gene(s) associated with antimicrobial resistance					Plasmid type
				Colistin	Aminoglycoside	Beta-lactam	Fluoroquinolone	Trimethoprim- sulfamethoxazole	
1000-*pmrB*Δ	25/09/2014	1	512	*pmrB* Δ386–402	*aph(3')-Ia*, *aadA2*, *aac(6')-Ib*, *aac(6')Ib-cr*	*bla*_KPC-3_, *bla*_OXA-9_, *bla*_SHV-11_	*oqxA*, *oqxB*, *aac(6')Ib-cr*	*sul1*, *dfrA12*	pKpQIL, pIncX3
1041-*mcr*	03/11/2014	40	512	*mcr1.2*	*aph(3')-Ia*, *aadA2*, *aac(6')-Ib: aac(6')Ib-cr*, *aac(3)-Iid*, *aadA5*, *strA*, *strB*	*bla*_KPC-3_, *bla*_OXA-9_, *bla*_SHV-11_	*oqxA*, *oqxB*, *aac(6')Ib-cr*	*sul1*, *sul2*, *dfrA12*	pKpQIL, pIncX4, pIncQ, pIncX3
1074-*mcr*/ pIncX3Δ	01/12/2014	67	512	*mcr1.2*	*aph(3')-Ia*, *aadA2*, *aac(6')-Ib*, *aac(6')Ib-cr*, *aac(3)-Iid*, *aadA5*, *strA*, *strB*	*bla*_KPC-3_, *bla*_OXA-9_, *bla*_SHV-11_	*oqxA*, *oqxB*, *aac(6')Ib-cr*	*sul1*, *sul2*, *dfrA12*	pKpQIL, pIncX4, pIncQ
1140-*mcr*	25/09/2015	365	512	*mcr1.2*	*aph(3')-Ia*, *aadA2*, *aac(6')-Ib: aac(6')Ib-cr*, *aac(3)-Iid*, *aadA5*, *strA*, *strB*	*bla*_KPC-3_, *bla*_OXA-9_, *bla*_SHV-11_	*oqxA*, *oqxB*, *aac(6')Ib-cr*	*sul1*, *sul2*, *dfrA12*	pKpQIL pIncX4, pIncQ
1303-*colS*			37			*bla*_SHV-11_			

a All isolates were recovered from rectal swab samples.

**TABLE 2:** Patterns of susceptibility to the antibiotics tested

Isolate	MIC (mg/liter)^*a*^													
	AMIKA	AMC	CFPM	CTX	CAZ	COL	ERTA	FOS	GN	IMI	MERO	TZP	TIG	STX
1000-*pmrB*Δ	>16 (R)	>8 (R)	>32 (R)	>4 (R)	>128 (R)	>8 (R)	>1 (R)	≤16 (S)	≤1 (S)	8 (I)	32 (R)	>128 (R)	0.5 (S)	>4 (R)
1041-*mcr*	16 (I)	>8 (R)	>32 (R)	>4 (R)	64 (R)	>8 (R)	>1 (R)	≤16 (S)	>4 (R)	16 (R)	32 (R)	>128 (R)	0.5 (S)	>4 (R)
1074-*mcr*/ pIncX3Δ	>16 (R)	>8 (R)	>32 (R)	>4 (R)	>128 (R)	>8 (R)	>1 (R)	≤16 (S)	>4 (R)	16 (R)	32 (R)	>128 (R)	0.5 (S)	>4 (R)
1303-*colS*	≤4 (S)	8 (S)	2 (S)	0.12 (S)	0.25 (S)	0.12 (S)	>1 (R)	NT	NT	2 (S)	2 (S)	4 (S)	0.5 (S)	2 (S)
1140-*mcr*	>16 (R)	>8 (R)	>32 (R)	>4 (R)	>128 (R)	>8 (R)	>1 (R)	≤16 (S)	>4 (R)	16 (R)	32 (R)	>128 (R)	0.25 (S)	>4 (R)

a AMIKA, amikacin; AMC, amoxicillin-clavulanic acid; CFPM, cefepime; CTX, cefotaxime; CAZ, ceftazidime; COL, colistin; ERTA, ertapenem; FOS, fosfomycin; GN, gentamicin; IMI, imipenem; MERO, meropenem; TZP, piperacillin-tazobactam; TIG, tigecycline; STX, trimethoprim-sulfamethoxazole; NT, not tested. The designations in parentheses indicate that the isolate was intermediate (I), resistant (R), or susceptible (S).

### wgMLST analysis.

Assemblies of the reads of isolates 1000-*pmrB*Δ, 1041-*mcr*, and 1074-*mcr*/pIncX3Δ resulted in draft genomes of 110 contigs and 5,701,465 bp, 89 contigs and 5,605,594 bp, and 95 contigs and 5,565,702 bp, respectively. The colR-KPC3-KP strains analyzed in the present study belonged to sequence type 512 (ST512). These isolates, together with 30 Klebsiella pneumoniae isolates from patients from a previous study (4), were selected for comparison by whole-genome multilocus sequence typing (wgMLST). The characteristics of the above-mentioned isolates are summarized in Table S1 in the supplemental material. There were no allelic mismatches between isolate 1000-*pmrB*Δ and isolate 1041-*mcr*, while there were two allelic mismatches between isolates 1041-*mcr* and 1074-*mcr*/pIncX3Δ, as shown in Fig. S1. Overall, the four isolates were isogenic, with at most 4 out of 4,884 alleles being different.

10.1128/mSphere.00551-19.1 FIG S1 Minimum-spanning tree based on allelic mismatch between 33 colR-KPC3-KP isolates. The spanning tree is based on 4,884 alleles analyzed pairwise, ignoring missing values. The red circle contains strains isolated from the same patient. No allelic mismatches between isolate 1000-*pmrB*Δ and isolate 1041-*mcr* were detected. Other isolates were collected in the same hospital and characterized in a previous study (BioProject accession number PRJEB19808). Download FIG S1, JPG file, 0.2 MB. Copyright © 2019 Giordano et al. 2019 Giordano et al. This content is distributed under the terms of the Creative Commons Attribution 4.0 International license.

10.1128/mSphere.00551-19.3 TABLE S1 Characteristics of the 30 Klebsiella pneumoniae isolates from a previous study (4). Nonsynonymous nucleotide mutations and their positions in the reference coding sequence are presented. *, a neutral mutation predicted not to cause functional changes to the protein; I, intermediate; R, resistant; S, susceptible. Download Table S1, DOCX file, 0.02 MB. Copyright © 2019 Giordano et al. 2019 Giordano et al. This content is distributed under the terms of the Creative Commons Attribution 4.0 International license.

### Growth curve analysis.

**FIG 2:**
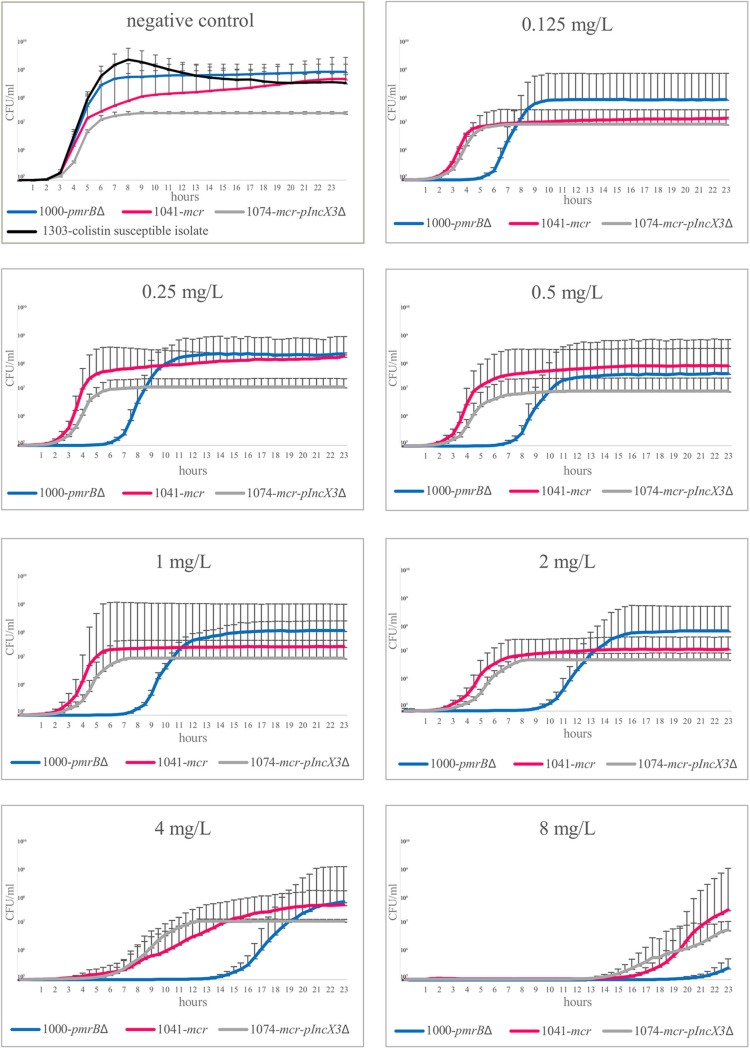
Growth curve experiments with isolates 1000-*pmrB*Δ, 1041-*mcr*, and 1074-*mcr*/pIncX3Δ. The mean and bandwidth of the growth of the three replicates measured every 30 min are presented. Without colistin exposure, isolate 1000-*pmrB*Δ, collected in September 2014, and isolate 1041-*mcr*, collected in November 2014, had identical growth characteristics (*P* ≤ 0.05). Isolate 1074-*mcr*/pIncX3Δ, collected in December 2014, seemed to grow slower in the negative control, but following colistin exposure, both isolate 1041-*mcr* and isolate 1074-*mcr*/pIncX3Δ showed a growth advantage compared to isolate 1000-*pmrBΔ*.

**FIG 3:**
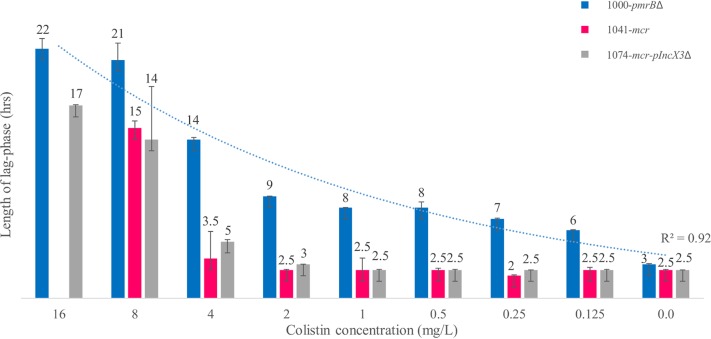
Analysis of the lag phase of isolates 1000-*pmrB*Δ, 1041-*mcr*, and 1074-*mcr*/pIncX3Δ. In the negative control, the leg phases of the isolates were identical (*P* ≤ 0.05), but the lag phase of isolate 1000-*pmrB*Δ increased exponentially following colistin exposure (*R*^2^ = 0.92), going from 3 to 22 h in the presence of colistin concentrations ranging from 0 to 16 mg/liter.

The isolates 1000-*pmrB*Δ, 1041-*mcr*, 1074-*mcr*/pIncX3Δ, and 1303-*colS* were selected for growth experiments. Isolates 1000-*pmrB*Δ and 1041-*mcr* showed no allelic mismatches by wgMLST; isolate 1041-*mcr* carried plasmid pIncX4-AOUP harboring the *mcr1.2* gene. The plasmid pIncX3-AOUP was not found in isolate 1074-*mcr*/pIncX3Δ. The growth curves of isolates 1000-*pmrB*Δ, 1041-*mcr*, and 1303-*colS* were similar in the absence of colistin; isolate 1074-*mcr*/pIncX3Δ showed reduced growth without colistin exposure. Colistin exposure altered the growth curves. As shown in Fig. 2, exposure of isolate 1000-*pmrB*Δ at a colistin concentration of 0.125 mg/liter increased the lag-phase time from 3 to 6 h. Isolates 1041-*mcr* and 1074-*mcr*/pIncX3Δ showed a reduced fitness cost according to their lag-phase times compared to the lag-phase time of isolate 1000-*pmrB*Δ at the same colistin concentration. Overall, at colistin concentrations of 0.125 mg/liter, 0.25 mg/liter, 0.5 mg/liter, 1 mg/liter, 2 mg/liter, 4 mg/liter, and 8 mg/liter, the lag phase of isolate 1000-*pmrB*Δ increased exponentially (R^2^ = 0.9205), going from 3 to 21 h, and was significantly longer than that of isolates 1041-*mcr* and 1074-*mcr*/pIncX3Δ (*P* ≤ 0.05) (Fig. 3). No lag-phase differences were found between isolates 1041-*mcr* and 1074-*mcr*/pIncX3Δ at colistin concentrations of 0.125 mg/liter, 0.25 mg/liter, 0.5 mg/liter, 1 mg/liter, 2 mg/liter, 4 mg/liter, and 8 mg/liter. However, at a colistin concentration of 16 mg/liter, isolate 1041-*mcr* did not grow.

We also performed the growth curves without cation adjustment (Fig. S2), and we noticed reduced lag-phase times for all isolates at medium to high levels of colistin exposure compared to those seen on the corresponding growth curves in experiments performed using cation-adjusted Mueller-Hinton broth, most likely because colistin loses its activity without cation adjustment.

10.1128/mSphere.00551-19.2 FIG S2 Analysis of the lag phase of isolates 1000-*pmrB*Δ, 1041-*mcr*, and 1074-*mcr*/pIncX3Δ performed without cation adjustment. In the negative control, the lag phases of isolates 1000-*pmrB*Δ, 1041-*mcr*, and 1074-*mcr*/pIncX3Δ were identical (*P* ≤ 0.05). The lag phase of isolate 1000-*pmrB*Δ remained the same following colistin exposure. In general, all isolates exhibited a reduced lag-phase time following colistin exposure compared to that on the corresponding growth curves determined using cation-adjusted Mueller Hinton broth. Download FIG S2, JPG file, 0.2 MB. Copyright © 2019 Giordano et al. 2019 Giordano et al. This content is distributed under the terms of the Creative Commons Attribution 4.0 International license.

We sequenced isolates 1041-*mcr* and 1074-*mcr*/pIncX3Δ after growth with colistin at concentrations of 4 mg/liter and 8 mg/liter. For these isolates, no spontaneous nucleotide mutations in the genes *mgrB*, *prmA*, *prmB*, *phoP*, and *phoQ* compared to the sequences before exposure were detected by whole-genome sequences analysis.

## DISCUSSION

We describe the persistent carriage of the *mcr-1.2* gene in the successful CP-KP lineage MLST ST512, isolated from a patient in Italy. The acquisition of the plasmid carrying *mcr-1.2* occurred under gentamicin treatment and was probably triggered by coselection of an associated plasmid carrying aminoglycoside resistance genes. This case provided the opportunity to study the genuine evolution of resistance and compare the fitness costs of different resistance mechanisms under colistin exposure in isogenic isolates.

In Europe, the *mcr-1* gene has been identified mainly in animal samples but rarely in human samples. Previous studies described the prevalence of *mcr-1* in animals, ranging from 0.5% to 13.5% (12). The oldest isolate reported dates back to 2005 and was an Escherichia coli strain from a calf in France (13). Human isolates have sporadically been reported from the Netherlands (14), Spain (15), England (16), Switzerland (17), and Italy (18). The majority of the infections are caused by E. coli and are related to the gastrointestinal tract. In addition, wound and bloodstream infections have also been reported (19).

Recent reports show the presence of *mcr* genes in carbapenemase-producing *Enterobacteriaceae*. The combination of *bla*_KPC_ and *mcr-1* has been described in an isolate from a German patient: a KPC-2-producing E. coli ST362 isolate causing a foot wound infection (19). Moreover, the interpatient spread of an E. coli isolate with the combination of the carbapenemase *ndm-5* and *mcr-1* has been described in patients from China (20). These reports underline the importance of surveillance for *mcr-1* in health care settings.

The genomic analysis presented in this study revealed that the first CP-KP isolate had a mutated *pmrB* and that the subsequent isogenic isolates harbored *mcr-1.2* but an intact *pmrB.* The isolate harboring the *mcr-1.2* gene showed reduced fitness costs compared to those for the isolate carrying the mutated *pmrB* gene in the presence of colistin concentrations that are reached *in vivo* (median concentration, 3.7 mg/liter; 5th and 95th percentiles, 0.7 and 12.5 mg/liter, respectively) (21). Recently, Tietgen and colleagues (22) showed that the expression of *mcr-1* is able to cause a fitness cost when carried by expression vectors. We showed that in the absence of colistin, isolates 1000-*pmrB*Δ and 1041-*mcr* and the colistin-susceptible CP-KP isolate had similar growth characteristics. The reduced growth costs of isolates 1041-*mcr* and 1074-*mcr*/pIncX3Δ could be explained according to the Harrison and Brockhurst plasmid paradox (23): even though plasmids represent a burden upon arrival in a new host, they also produce some beneﬁts, improving the cost over time through compensatory mutations in the plasmid and the host chromosome. Interestingly, we detected a mutated *pmrB* gene in the first isolate, but the wild-type *pmrB* gene was found in subsequent isogenic isolates. Attempts were made to look for possible *pmrB* recombination sites in the genomes both on the chromosome and on mobile genetic elements. The region adjacent to the 17-nucleotide deletion in *pmrB* was investigated. The deleted region and several nucleotides surrounding the deletion were found in the chromosome on two different contigs; the first part was in the *ppiB* gene, coding for a cysteine tRNA ligase, and the second part was in an untranslated region. Furthermore, the deleted region was found on the pIncX3-AOUP plasmid; the first part was found in the *parB* gene, and the second part was found in a transposase. Therefore, we cannot rule out with certainty the possibility of the repair of *pmrB* by recombination events, but it seems unlikely. Conceivably, undetected clonal variants with an intact *pmrB* gene were already present in the first culture and acquired the *mcr-1.2*-carrying plasmid later on.

The origin of the colR-KPC3-KP strain harboring *mcr-1.2* remains unknown. Gentamicin therapy might have coselected these multidrug-resistant bacteria, as shown also by the acquisition of the plasmid pIncQ-AOUP. The plasmid pIncQ-AOUP is a nonconjugative broad-host-range plasmid conferring gentamicin resistance. Both pIncQ-AOUP and pIncX4-AOUP were newly acquired. These are coresident plasmids: pIncX4-AOUP provides the conjugative system for the mobilization of the pIncQ-AOUP plasmid (24). The coresidency of these plasmids may explain their selective advantage under gentamicin pressure, even though the patient was not under colistin treatment when plasmid pIncX4-AOUP was acquired. The source of these plasmids is unknown. Farm animals may be an important reservoir of the *mcr*-carrying plasmids, since their prevalence is common in animals and retail meat but rare in humans (25). The nosocomial dissemination of *mcr*-carrying plasmids might also be taken into consideration in our case. However, this seems less likely, since the molecular evaluation of 30 clinical isolates of Klebsiella pneumoniae collected at the hospital during 2015 and 2016 revealed no other strains harboring *mcr-1.2*, while the most common mutation associated with resistance to colistin affected the *mgrB* gene (4).

In conclusion, our study showed a successful prolonged human colonization by a colR-KPC3-KP isolate harboring *mcr-1.2*. The coselection of resistance plasmids under antibiotic therapy has contributed to the maintenance of this microorganism through the acquisition of new resistance genes. The isolates 1041-*mcr* and 1074-*mcr*/pIncX3Δ showed reduced fitness costs compared to those of the mutated *pmrB*Δ isolate. Both coselection and the reduced fitness costs of the resistance mediated by plasmids may contribute to the ability of the microorganism to persist despite antibiotic treatment. Therefore, it is important to prevent the spread of *mcr*-carrying plasmids and their coresiding multiresistance plasmids.

## MATERIALS AND METHODS

### Culture and characterization of isolates.

In a previously characterized library of 30 CP-KP isolates, collected between 2014 and 2015 in the Azienda Ospedaliero-Universitaria Pisana, deposited at the European Nucleotide Archive (BioProject accession number PRJEB19808), we identified a *mcr-1.2*-positive strain (isolate 1140-*mcr*), which was isolated in September 2015 from a pediatric hematology patient who was frequently hospitalized at the Azienda Ospedaliero-Universitaria Pisana in Pisa, Italy. We set out to perform a retrospective investigation of the microbiological results for this patient from 2014 to 2017 and sequenced all colR-KPC3-KP isolates.

Over this period, in routine diagnostics, rectal swab specimens were cultured on Chapman agar, Sabouraud agar, and MacConkey agar (Thermo Fisher Scientific, Oxoid) supplemented with meropenem disks (10 μg; Bio-Rad) and incubated at 37°C for 24 h. Colonies suspected to be CP-KPs were identified using a matrix-assisted laser desorption ionization–time of ﬂight mass spectrometer (MALDI-TOF MS; Bruker Daltonics, Bremen, Germany). Antimicrobial susceptibility tests were performed using a broth microdilution assay (SensiTitre; Thermo Fisher Scientific). The MIC results were interpreted using EUCAST guidelines (v5.0).

### Genome assembly and analysis.

The colR-KPC3-KP colonies were stored in brain heart infusion broth (BHIB) and 10% glycerol at −80°C for DNA extraction. Total genomic DNA was extracted using an UltraClean microbial DNA isolation kit (MoBio Laboratories), according to the manufacturer’s instructions. The concentration and purity of the extracted DNA were determined by use of a Qubit (v2.0) fluorometer and a double-stranded DNA BR assay kit (Life Technologies). The DNA library was prepared using a Nextera XT library preparation kit (v01; Illumina), according to the manufacturer’s instructions, and then run on a MiSeq system (Illumina) to generate paired-end 250-bp reads. *De novo* assembly was performed by use of the CLC Genomics Workbench (v9.5.2) (Qiagen) after quality trimming (quality score [Qs] ≥ 20). Multilocus sequence types were assessed using SeqSphere (v3.4.0) software (Ridom GmbH). An *ad hoc* scheme based on the core and acquired genomes was created using SeqSphere software. A minimum-spanning tree based on allelic mismatch (4,884 columns) was constructed. We preferred this whole-genome MLST scheme over the core genome MLST to achieve a higher discriminative power in distinguishing the patient’s isolates from the clonally related isolates of the ongoing outbreak with KPC-3-producing K. pneumoniae in Italy. The assembled genomes were uploaded to web tools called ResFinder (v2.1) (26) to identify acquired resistance genes and PlasmidFinder (v1.3) (27) to detect the replicons of the plasmids. The DNA sequences of *mgrB* (GenBank accession number KF852760), *phoQ* (GenBank accession number CP003200.1; nucleotides c2077641 to 2076175), *phoP* (GenBank accession number NC_016845.1; nucleotides c2078312 to 2077641), *pmrA* (GenBank accession number HG794234.1; nucleotides 3372 to 4043), and *pmrB* (HG794234.1; nucleotides 2271 to 3368) were used as references for detecting gene mutations associated with colistin resistance. Provean online software (28) was used to predict whether an amino acid substitution had an impact on the biological function of the proteins. The contigs of the plasmid sequences of interest, detected by PlasmidFinder, were manually reconstructed and analyzed using the Artemis comparison tool (ACT) (29) and related tools. Plasmids were mapped against complete reference sequences of pRSF1010_SL1344 (GenBank accession number HE654726), pKpQIL-10 (GenBank accession number KJ146687), pNDM_MGR194 (GenBank accession number NC_022740), and pMCR1.2-IT (GenBank accession number KX236309). The BLAST Ring Image Generator (BRIG) was used to display plasmid sequence comparisons (30).

### Growth curve experiments.

Overall, 162 growth curves were analyzed, using an HB&L instrument (Alifax S.r.l.), for isolates 1000-*pmrB*Δ, 1041-*mcr*, and 1074-*mcr*/pIncX3Δ. The colistin-susceptible K. pneumoniae isolate 1303-*colS*, from an unrelated patient, was used as a control. The instrument is certified to assess growth curves based on a light-scattering technique that reliably detects microbial growth in fluid samples. It calculates real-time growth curves and bacterial counts (in numbers of CFU per milliliter) following a patented algorithm (31). Growth curves were performed off-label in triplicate and with different concentrations of colistin sulfate (0.125 mg/liter, 0.25 mg/liter, 0.5 mg/liter, 1 mg/liter, 2 mg/liter, 4 mg/liter, 8 mg/liter, 16 mg/liter; Discovery Fine Chemicals) in cation-adjusted Mueller-Hinton broth (Merlin). The growth curves were also performed without cation adjustment. For each strain, a 0.5 McFarland suspension was prepared, and then serial dilutions were performed to obtain an initial inoculum of 5 × 10^5^ CFU/ml. The exact inocula were confirmed by plating the serial dilutions of the cultures. After reading of a blank to set the analytical value of 0, the scattering units (refracted light) were measured every 5 min for 24 h at 37°C, detecting only viable, replicating bacteria. Negative-control curves, performed without colistin sulfate, were performed and used as comparators. Isolates 1041-*mcr* and 1074-*mcr*/pIncX3Δ were assessed for spontaneous mutations in *pmrB*, *mgrB*, *phoP*, *phoQ*, and *prmA* by whole-genome sequencing after growth in cation-adjusted Mueller-Hinton broth including 4 mg/liter and 8 mg/liter colistin for 24 h.

### Data availability.

The sequences of the isolates determined in this study are available from the European Nucleotide Archive under study accession number PRJEB25114.
