# Supplementary figures and images for: Reduced Fitness Costs of mcr-1.2 Compared to Mutated pmrB in Isogenic Colistin-Resistant KPC-3-Producing Klebsiella pneumoniae
Source: mSphere. 2019 Nov 6;4(6):e00551-19. doi: 10.1128/mSphere.00551-19 (PMC6835208; doi:10.1128/mSphere.00551-19)

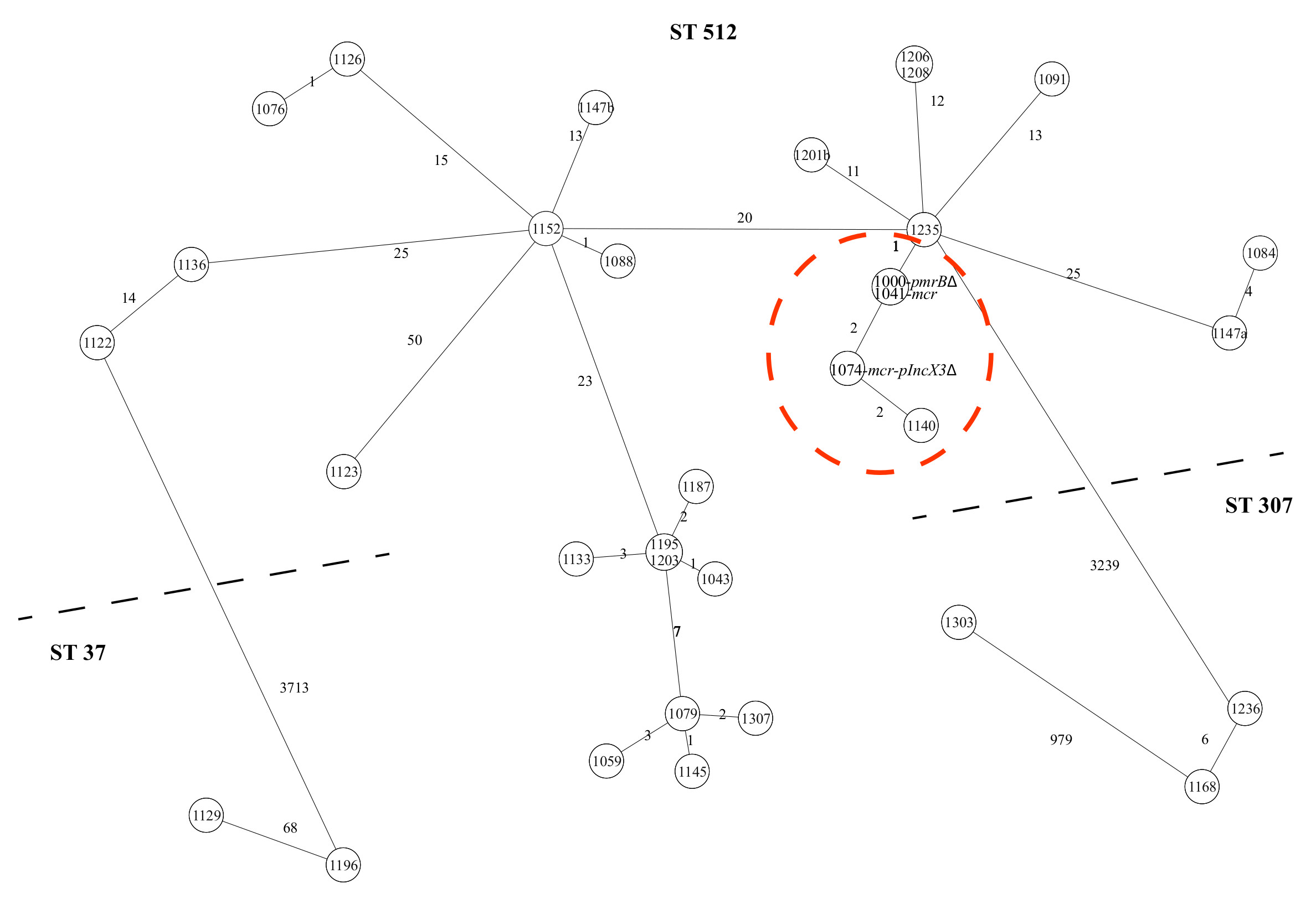

Supplement: FIG S1 [file mSphere.00551-19-sf001.jpg]

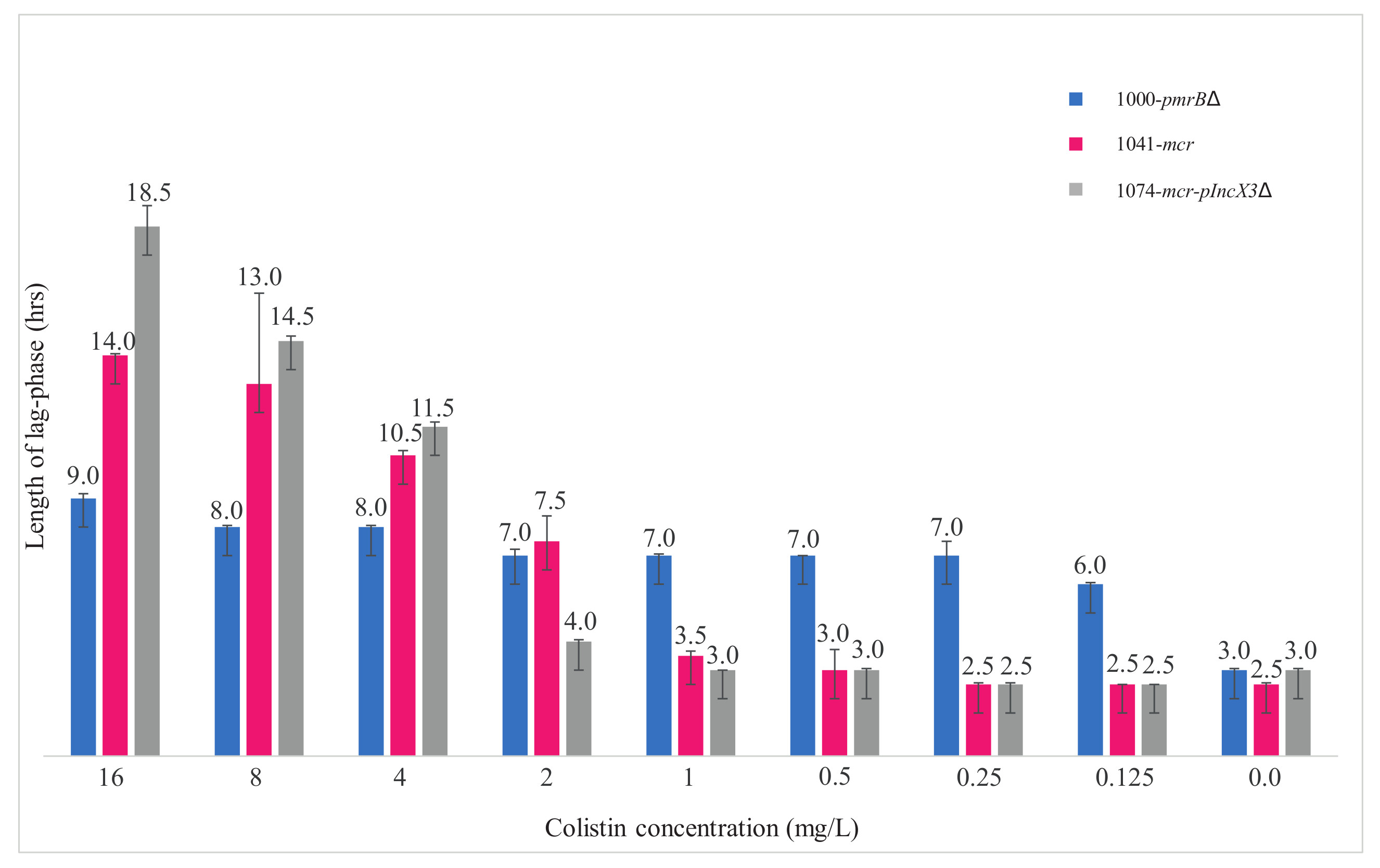

Supplement: FIG S2 [file mSphere.00551-19-sf002.jpg]
