# Supplementary material for: Reduced Fitness Costs of mcr-1.2 Compared to Mutated pmrB in Isogenic Colistin-Resistant KPC-3-Producing Klebsiella pneumoniae
Source: mSphere. 2019 Nov 6;4(6):e00551-19. doi: 10.1128/mSphere.00551-19 (PMC6835208; doi:10.1128/mSphere.00551-19)
Supplement: TABLE S1 [file mSphere.00551-19-st001.docx]

| **Isolate** | **isolation date** | **MLST** | **MIC COL**  **(mg/L)** | **mutations associated with colistin resistance** | **MIC MER (mg/L)** | **beta-lactam** |
| --- | --- | --- | --- | --- | --- | --- |
| 1084 | 07/2015 | 512 | >8 R | - | >64 R | *KPC*-3; *TEM*-1A; *OXA*-9; *SHV*-11 |
| 1091 | 07/2015 | 512 | >8 R | - | 64 R | *KPC*-3; *TEM*-1A; *OXA*-9; *SHV*-11 |
| 1122 | 08/2015 | 512 | >8 R | Δ109/119 *mgrB*; *PhoP* T602C; *PmrB* 697 (nnt), G245T* | 2 S | *SHV*-11 |
| 1123 | 02/2015 | 512 | 4 R | *PhoP* C55A* | 32 R | *KPC*-3; *TEM*-1A; *OXA*-9; *SHV*-11 |
| 1129 | 09/2015 | 307 | 4 R | *PmrA* G121A; *PmrB* G385C, A637C*, G766C* | 32 R | *KPC*-3; *TEM*-1A; *OXA*-9; *SHV*-28; *CTX-M-*15;  *OXA*-1 |
| 1145 | 10/2015 | 512 | >8 R | Δ109/119 *mgrB* | >64 R | *KPC*-3; *TEM*-1A; *OXA*-9; *SHV*-11 |
| 1147a | 02/2015 | 512 | >8 R | - | 64 R | *KPC*-3; *TEM*-1A; *OXA*-9; *SHV*-11 |
| 1196 | 04/2015 | 307 | >8 R | *PmrA* G121A; *PmrB* G245T*, C637A*, G766C* | 4 I | *KPC*-3; *TEM*-1A; *OXA*-9; *SHV*-28; *CTX-M-*15;  *OXA*-1 |
| 1201b | 04/2015 | 512 | 4 R | - | >64 R | *KPC*-3; *TEM*-1A; *OXA*-9; *SHV*-11 |
| 1206 | 12/2015 | 512 | >8 R | ISL3 133 | >64 R | *KPC*-3; *OXA*-9; *SHV*-11 |
| 1043 | 05/2015 | 512 | >8 R | Δ109/119 *mgrB* | 64 R | *KPC*-3; *TEM*-1A; *OXA*-9; *SHV*-11 |
| 1059 | 06/2015 | 512 | >8 R | Δ109/119 *mgrB* | 64 R | *KPC*-3; *TEM*-1A; *OXA*-9; *SHV*-11 |
| 1076 | 06/2015 | 512 | >8 R | Δ109/119 *mgrB* | 64 R | *KPC*-3; *TEM*-1A; *OXA*-9; *SHV*-11 |
| 1079 | 06/2015 | 512 | >8 R | Δ109/119 *mgrB* | 64 R | *KPC*-3; *TEM*-1A; *OXA*-9; *SHV*-11 |
| 1088 | 07/2015 | 512 | >8 R | Δ109/119 *mgrB* | 64 R | *KPC*-3; *TEM*-1A; *OXA*-9; *SHV*-11 |
| 1126 | 09/2015 | 512 | >8 R | Δ109/119 *mgrB* | >64 R | *KPC*-3; *TEM*-1A; *OXA*-9; *SHV*-11 |
| 1133 | 02/2015 | 512 | >8 R | Δ109/119 *mgrB* | 64 R | *KPC*-3; *TEM*-1A; *OXA*-9; *SHV*-11 |
| 1136 | 09/2015 | 512 | >8 R | Δ109/119 *mgrB* | >64 R | *KPC*-3; *OXA*-9; *SHV*-11 |
| 1152 | 11/2015 | 512 | >8 R | Δ109/119 *mgrB* | 16 R | *KPC*-3; *TEM*-1A; *OXA*-9; *SHV*-11 |
| 1168 | 07/2015 | 37 | >8 R | *mgrB* A7T Stop codon | 64 R | *KPC*-3; *VIM*-1; *OXA*-9; *SHV*-11 |
| 1187 | 03/2015 | 512 | >8 R | Δ109/119 *mgrB* | 64 R | *KPC*-3; *TEM*-1A; *OXA*-9; *SHV*-11 |
| 1195 | 04/2015 | 512 | >8 R | Δ109/119 *mgrB* | 64 R | *KPC*-3; *TEM*-1A; *OXA*-9; *SHV*-11 |
| 1203 | 04/2015 | 512 | >8 R | Δ109/119 *mgrB* | 64 R | *KPC*-3; *TEM*-1A; *OXA*-9; *SHV*-11 |
| 1140 | 09/2015 | 512 | >8 R | *mcr-1.2* | 32 R | *KPC*-3; *OXA*-9; *SHV*-11 |
| 1147b | 10/2015 | 512 | >8 R | Δ109/119 *mgrB* | 64 R | *KPC*-3; *OXA*-9; *SHV*-11 |
| 1208 | 01/2016 | 512 | >8 R | ISL3 133 | 64 R | *KPC*-3; *TEM*-1A; *OXA*-9; *SHV*-11 |
| 1235 | 02/2016 | 512 | >8 R | IS5 75 | 64 R | *KPC*-3; *TEM*-1A; *OXA*-9; *SHV*-11 |
| 1236 | 02/2016 | 37 | >8 R | *mgrB* A7T Stop codon; *PmrA* C121T | >64 R | *KPC*-3; *VIM*-1; *TEM*-1A; *OXA*-9; *SHV*-11 |
| 1303 | 05/2016 | 512 | >8 R | - | 0.25 S | *SHV*-11 |
| 1307 | 06/2016 | 37 | >8 R | Δ109/119 *mgrB* | 64 R | *KPC*-3; *TEM*-1A; *OXA*-9; *SHV*-11 |
